# Medical Vision-Language Models: Existing Technologies, Clinical Applications and Future Directions

**DOI:** 10.3390/s26133998

**Published:** 2026-06-24

**Authors:** Le Zou, Mengyu Ma, Jun Li, Hao Chen, Shuang Peng

**Affiliations:** College of Electronic Science and Technology, National University of Defense Technology, No. 109 Deya Road, Kaifu District, Changsha 410073, China; zoule41@nudt.edu.cn (L.Z.); mamengyu10@nudt.edu.cn (M.M.); hchen@nudt.edu.cn (H.C.); pengshuang08@nudt.edu.cn (S.P.)

**Keywords:** vision-language model, medical image analysis, artificial intelligence, multi-modal learning, clinical application

## Abstract

Medical image analysis is a cornerstone of modern healthcare, yet conventional single-modal deep learning often struggles with the unique physical constraints and structural variability inherent in data acquired from diverse medical sensors. Recently, Vision-Language Models (VLMs) have sparked a paradigm shift by bridging the semantic gap between visual sensor signals and clinical narratives. Following the PRISMA guidelines, 167 representative studies are systematically synthesized in this review to provide a comprehensive roadmap of VLM technological evolution and clinical utility. First, rather than treating VLMs as generic feature extractors, their underlying mechanisms are uniquely distilled into seven core operational principles, which are then explicitly mapped to downstream applications such as few-shot diagnosis, prompt-driven segmentation, and multi-task foundation models. To facilitate intuitive evaluation, a rigorous quantitative cross-comparison of current benchmark architectures is presented. Crucially, this review goes beyond highlighting successes by critically assessing prevalent clinical bottlenecks, including zero-shot segmentation failures, multi-modal hallucinations in diagnosing rare diseases, and the prohibitive computational complexity associated with 3D volumes and gigapixel whole slide images. Finally, a novel, forward-looking framework is proposed: the transition from static “image-text alignment” to dynamic “multi-source sensor-driven intelligence”. By addressing both physical sensor constraints and algorithmic limitations, this survey offers actionable insights for developing trustworthy, sensor-aware clinical diagnostic agents.

## 1. Introduction

With the rapid evolution of artificial intelligence (AI), the convergence of computer vision and natural language processing has emerged as a critical frontier. Vision-language models (VLMs), as a representative multi-modal paradigm, have garnered extensive attention for their ability to establish deep cross-modal interactions [1]. Driven by breakthroughs in foundation models—ranging from encoder-based architectures like BERT [2] to generative large language models (LLMs) like GPT [3]—VLMs have evolved beyond simple feature fusion. They now enable sophisticated semantic alignment and mutual understanding between visual and linguistic modalities [4]. This paradigm shift not only enhances basic capabilities such as image recognition but also unlocks advanced potentials in cross-modal retrieval, reasoning, and content generation, positioning VLMs as a cornerstone for intelligent signal and information processing across diverse sensing domains [5].

In the medical domain, the accurate interpretation of complex imaging data is a prerequisite for critical clinical workflows [6]. Technically, these images are the digitized outputs of sophisticated sensing hardware—such as radio-frequency (RF) coils in MRI, X-ray photon detectors in CT, and piezoelectric transducers in ultrasound systems—which capture high-dimensional physical signals from the human body [7]. Crucially, the distinct physical mechanisms of these sensors impose unique data constraints on the architectural design of VLMs. For instance, CT X-ray detectors generate dense, isotropic 3D voxel grids with standardized Hounsfield units, strictly requiring VLMs to manage massive token sequences and preserve high-fidelity structural semantics without exceeding memory constraints. Conversely, MRI RF coils produce multi-parametric signals (e.g., T1, T2, FLAIR) characterized by non-standardized intensity distributions and complex frequency-domain noise, which necessitates VLMs to possess robust intra-modal normalization and cross-sequence alignment capabilities. Furthermore, ultrasound piezoelectric transducers capture real-time, operator-dependent dynamic echoes afflicted by heavy speckle noise, demanding VLMs to incorporate temporal sequence modeling and noise-robust attention mechanisms to achieve effective prompt-driven reasoning. However, conventional medical image analysis predominantly relies on single-modal feature extraction [8]. These approaches often struggle with the inherent high dimensionality and structural variability of sensor-acquired medical data (e.g., distinguishing subtle lesions), leading to limited generalizability and a lack of interpretability in clinical practice [9]. This highlights the urgent need for a more holistic approach. VLMs offer an innovative solution by bridging the “semantic gap” between pixel-level visual signals and high-level clinical narratives [10]. By integrating diverse modalities—ranging from X-Ray, CT, MRI [11], ultrasound [12], and pathological images [13] to radiology reports [14] and electronic medical records [15] —VLMs not only capture comprehensive pathological associations but also enhance the transparency of decision-making by aligning sensing findings with clinical reasoning [16,17].

Technically, medical VLMs are witnessing a transition from task-specific fusion strategies to universal representation learning. Fueled by large-scale medical datasets acquired from diverse sensing modalities [18], recent studies leverage masked modeling [19], contrastive learning [20], and cross-modal matching [21] to construct pre-trained foundation models. These models exhibit strong few-shot and zero-shot capabilities, allowing them to adapt to downstream tasks via mechanisms like prompt engineering, significantly reducing the reliance on massive labeled data.

**Figure 1:**
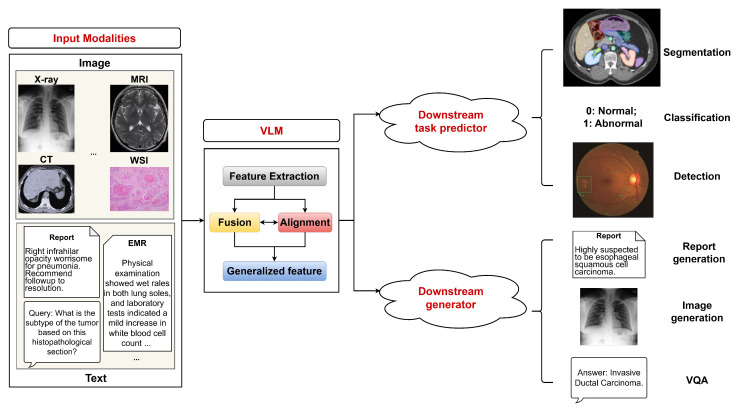
Clinical application landscape of VLMs.

Figure 1 illustrates the current landscape, covering both discriminative tasks (diagnosis [22], segmentation [23], and detection [24]) and generative tasks (report generation [25], Visual Question Answering (VQA) [26], and targeted generation of medical images for clinical training and simulation [27]).

VLM-based approaches for medical image analysis have attracted increasing interest, with related publications expanding rapidly in recent years [28]. This work presents a thorough survey of how VLMs are applied across medical image analysis tasks. The key contributions of this paper are summarized as follows:

- A broad review of VLM-driven methods for medical image analysis is presented. Over 150 representative studies are discussed, covering the evolution from early fusion paradigms to today’s widely adopted large-scale models.
- Drawing on extensive prior work, seven key properties of VLMs are distilled, and how each property supports different downstream medical imaging tasks is examined, thereby clarifying the practical role of VLMs in this domain.
- Three major challenges faced by current research—interactivity, trustworthiness, and multi-source sensor integration—are identified, and corresponding future directions are outlined. This includes a novel perspective on transitioning from static imaging to dynamic sensing ecosystems, which offers actionable guidance for future studies in the sensor-based AI field.

To provide a systematic perspective on this rapidly evolving field, this paper is structured as follows: Section 2 outlines the literature search methodology and taxonomy. Section 3 clarifies the distinction of this paper from existing reviews and the core operational principles of VLMs. Section 4 explores the core applications of VLMs in medical imaging in detail. Section 5 critically assesses existing challenges and identifies three key future directions. Section 6 concludes the paper.

## 2. Literature Search and Taxonomy Definition

### 2.1. Search Strategy and Selection Process

To ensure a rigorous and comprehensive review of VLMs in medical image analysis, a structured literature search was conducted across multiple academic databases covering the period from 2020 to 2025. Following the Preferred Reporting Items for Systematic Reviews and Meta-Analyses (PRISMA) guidelines, the complete details of the search methodology, including the databases consulted, specific Boolean query combinations, and strict inclusion/exclusion criteria, are presented in Table 1.

**Figure 2:**
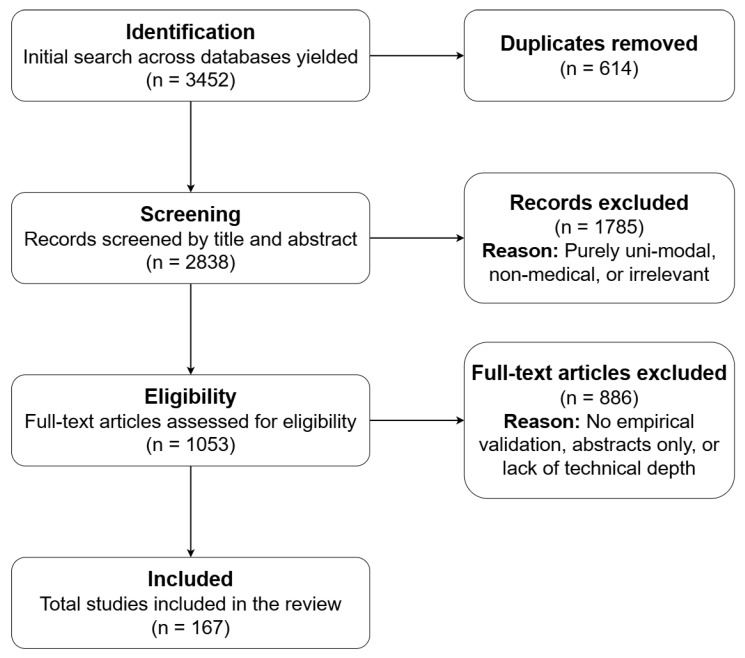
PRISMA flow diagram illustrating the literature search and selection process.

To clarify the literature screening process and ensure methodological transparency, Figure 2 illustrates the PRISMA flow diagram. The initial database search yielded 3452 candidate records. After removing 614 duplicates, 2838 unique articles underwent title and abstract screening. This phase led to the exclusion of 1785 papers that were purely uni-modal, non-medical, or irrelevant to the review’s scope. Subsequently, the remaining 1053 full-text articles were comprehensively assessed for eligibility. During this rigorous evaluation, 886 papers were excluded due to a lack of empirical validation, being conference abstracts without full-text availability, or lacking sufficient technical depth. Ultimately, a total of 167 representative studies were selected for final inclusion in this review.

Ultimately, 167 representative papers were selected for inclusion. It is important to note that according to the PRISMA 2020 guidelines, the final repository includes highly relevant studies identified through database searching as well as cutting-edge articles (including 2026 Early Access publications and Preprints) incorporated via expert peer-review recommendations. The final inclusion was strictly driven by three core metrics: (1) Relevance and Novelty: Articles introducing groundbreaking VLM architectures, or advanced theoretical algorithms from broader sensing domains (e.g., cross-lingual fusion, geometric optimization) that offer vital methodological support for medical tasks; (2) Taxonomy Fit: Studies that perfectly exemplify the seven operational principles defined in our taxonomy (Section 3); and (3) Impact: High citation counts for earlier foundational works and top-tier venue publications for the latest advancements.

### 2.2. Taxonomy Definition

**Figure 3:**
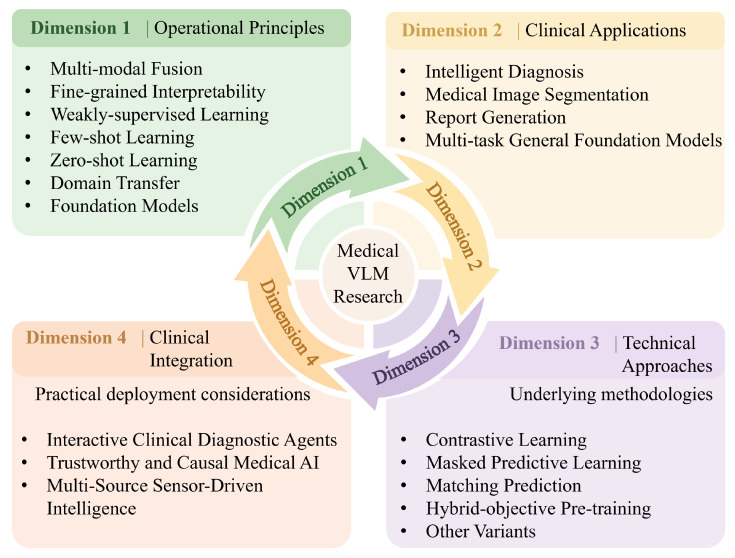
A hierarchical taxonomy of medical VLM research.

To systematically construct a clear roadmap of medical VLM research, a comprehensive taxonomy is proposed and presented as a radial framework in Figure 3. This hierarchical and visual taxonomy serves as the blueprint for the subsequent detailed discussions in Section 4, Section 5 and Section 6.

## 3. Characteristics of VLMs in Medical Image Analysis

### 3.1. Distinction from Existing Reviews

While several recent reviews have explored the intersection of AI and medical imaging, this work distinguishes itself by providing a deeply clinical and task-oriented perspective specifically tailored to VLMs. While previous surveys have primarily focused on the algorithmic architectures of multi-modal models [29] or the general use of foundation models in healthcare [30,31], they often overlook the fine-grained nuances of how VLMs specifically tackle clinical bottlenecks such as zero-shot inference for rare diseases, prompt-driven segmentation, and multi-grained report generation. Furthermore, unlike existing literature that mainly treats VLMs as feature extractors [32], this review systematically categorizes VLM characteristics into seven distinct operational principles (see Section 3.2) and rigorously explores the recent paradigm shift toward medical multi-task general foundation models.

### 3.2. Core Operational Principles of VLMs

In the clinical practice of medical image analysis, VLMs are predominantly applied to a series of core clinical-aided tasks, including medical image classification, image segmentation, medical report automatic generation, medical VQA, object detection, and medical image generation. The distinctive applications and characteristics of VLMs in medical image analysis can be concisely summarized into seven key operational principles, as presented in Table 2.

## 4. Application of VLMs in Medical Image Analysis

**Figure 4:**
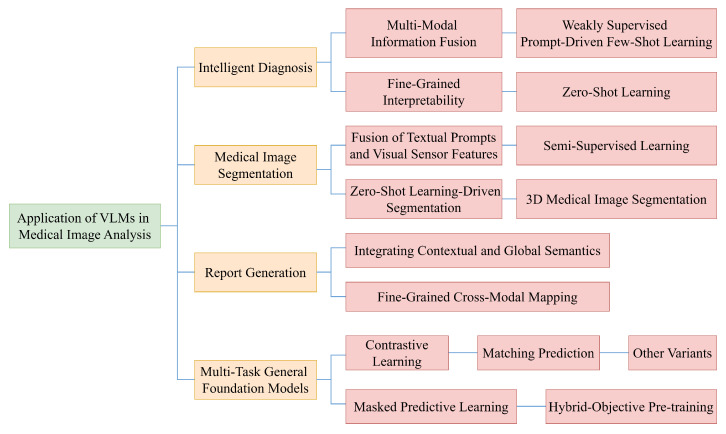
The taxonomy of VLM applications in medical image analysis.

Recent advances in VLMs have reshaped medical image analysis by enabling unified reasoning over visual evidence and clinical language. Beyond extracting discriminative visual features, VLMs integrate free-text reports, structured findings, and prompt-driven medical concepts, which supports not only routine diagnostic recognition but also more explainable, data-efficient, and generalizable clinical decision support. Accordingly, VLM applications can be divided into four perspectives: (i) medical image diagnosis; (ii) medical image segmentation; (iii) report generation; and (iv) multi-task foundation models (as shown in Figure 4).

### 4.1. Intelligent Algorithms for Medical Image Diagnosis

Medical image diagnosis, the primary objective of which is to improve the performance of disease classification and grading, holds the broadest clinical demand. In supervised classification, VLMs boost diagnostic accuracy through multi-modal fusion and guarantee interpretability by extracting fine-grained features. In unsupervised scenarios, VLMs focus on achieving few-shot diagnosis with the aid of weakly supervised prompts as well as zero-shot diagnosis.

#### 4.1.1. Multi-Modal Information Fusion in Medical Image Diagnosis

A key application of VLMs in medical image diagnosis lies in multi-modal fusion, which serves to enhance diagnostic accuracy and is well-suited for the comprehensive assessment of complex diseases [52]. For example, it involves fusing pathological section images, imaging examination data, clinical electronic medical record texts, and testing data to provide decision support for formulating precise cancer treatment plans [53]; in the diagnosis of cardiovascular diseases, it integrates ultrasound, CTA, and electrocardiogram data to improve the accuracy of vascular stenosis assessment [54].

Efficient multi-modal diagnosis necessitates a dual synergy: the robust extraction of pathological features from individual modalities and the precise alignment of these features within a unified semantic space. To capture diagnostic cues across varied granularities, hierarchical architectures have been introduced. For instance, the ViLa-MIL [33] framework employs a dual-tier multi-instance strategy to synchronize patch-level and whole-slide-level semantics, utilizing context-aware decoders and prototype-based aggregation to refine lesion-related representations. Complementary to spatial scaling, multi-frequency analysis has proven instrumental in uncovering subtle, implicit cross-modal correlations that traditional single-frequency extractors might overlook [22]. Furthermore, to manage the inherent complexity of multi-source sensor data, advanced theoretical frameworks from other imaging domains—such as regularized subspace classification methods originally designed for hyperspectral data [55]—can be seamlessly adapted. This cross-disciplinary adaptation strengthens the theoretical foundation for feature alignment when handling complex medical imaging modalities.

Furthermore, since clinical narratives often provide explicit spatial and morphological descriptors (e.g., specific lesion sites and contours), attention-driven mechanisms are frequently utilized to integrate these textual priors into the visual pipeline. This allows for a more flexible and interactive fusion of visual evidence and textual insights during the training phase [56]. Beyond raw text processing, the injection of structured medical expertise further optimizes diagnostic accuracy. This can be achieved by constructing knowledge graphs from clinical dialogues to guide semantic matching [57], or by leveraging domain-specific textual corpora to fortify the discriminative power of pathological image classifiers [58]. Such text-guided visual paradigms effectively demonstrate that incorporating external linguistic knowledge can significantly compensate for the limitations of single-modal visual analysis.

#### 4.1.2. Interpretability of Medical Image Diagnosis

A primary barrier to the clinical integration of deep learning is the “opaque” nature of traditional neural networks [59]. Relying solely on numerical probability scores is insufficient for high-stakes clinical environments, as it fails to provide the underlying rationale required for physician validation. To address this, VLMs have introduced two major paradigms to foster transparency: visual evidence grounding and semantic concept disentanglement.

1. Visual Evidence Grounding: This approach transitions the model from providing a singular label to generating spatially-aware justifications. By aligning disease-specific text embeddings with localized visual features [60], VLMs can produce high-fidelity heatmaps that highlight suspicious regions. For instance, the RC-TPL framework [35] optimizes this by extracting clinical descriptors and anatomical locations from radiology reports, thereby anchoring diagnostic conclusions to tangible visual evidence. Similarly, hierarchical fine-tuning strategies can be employed to enrich the textual context, allowing tools like Grad-CAM to visualize the diagnostic focus of pre-trained models more accurately [61].

2. Semantic Concept Disentanglement: This strategy, often referred to as “concept bottlenecks”, bridges the gap between raw pixels and diagnostic logic by introducing intermediate, medically-relevant attributes [62,63]. Rather than a direct image-to-class mapping, models are designed to first recognize discrete clinical features—such as the morphology, color, and distribution of skin lesions—and then derive the final diagnosis from these transparent “building blocks”. Research has demonstrated that visual activation scoring can effectively filter these concepts to balance performance with clarity [36]. Furthermore, for complex tasks like lung nodule assessment, contrastive learning has been utilized to align intermediate representations with benign or malignant attribute categories, ensuring that the model’s logic remains consistent with human expertise [37].

Such interpretability is vital for the clinical verification of high-risk procedures. In neuro-oncology, explicitly delineating tumor margins [64] can provide indispensable guidance for surgical resection planning. Moreover, in sensitive fields such as pediatrics or obstetrics, providing a clear “reasoning chain” is essential for fostering clinician trust and accelerating the practical translation of intelligent diagnostic systems into bedside care [65].

#### 4.1.3. Weakly Supervised Prompt-Driven Few-Shot Medical Image Diagnosis

In disease screening scenarios, a large volume of image data can be acquired, while sufficient annotations for disease categories remain scarce [66]. VLMs enable few-shot diagnosis by leveraging large-scale unlabeled data and weakly supervised text prompts [67,68], with the specific implementation pathway as follows:

By leveraging the vast prior knowledge within pre-trained VLMs, raw and unannotated medical observations can be automatically mapped to diverse textual descriptors. This process transforms linguistic metadata into weak supervision, which is instrumental in refining the classification of data acquired from various clinical sensors [38]. This approach is capable of addressing the issue of insufficient image data for rare diseases—for example, in the image diagnosis of rare cerebral genetic diseases and special types of tumors, efficient diagnostic models can be constructed by combining a small amount of labeled data with weakly supervised text prompt learning. Additionally, it is applicable to primary medical institutions or remote areas, facilitating accurate image diagnosis under the constraint of limited data resources.

In relevant research, Liang et al. [39] utilized text reports as weak supervision and performed model fine-tuning on small-scale annotated datasets. To accommodate the intricate nature of medical records and their rigid formats, Keicher et al. [41] introduced FlexR. This few-shot methodology relies on contrastive learning to automatically construct specific textual prompts corresponding to various clinical findings. In the context of pathology, Qu et al. [42] tackled Whole Slide Image (WSI) classification with limited weak supervision by introducing a dual-tier prompting strategy. Specifically, they utilized GPT-4 to synthesize both instance- and bag-level (package-level) prompts. Furthermore, to enhance unsupervised vision-language pre-training, Zheng et al. [40] incorporated a weakly supervised prompting mechanism, where visual categorical information is exploited to drive the optimization of class-aware representations within the text prompts.

#### 4.1.4. Open-Vocabulary Inference and Zero-Shot Diagnostic Generalization

A transformative capability of VLMs is their aptitude for zero-shot inference, which allows for the identification of unseen pathologies without the need for task-specific retraining. This is particularly vital for responding to emerging infectious diseases or screening for rare clinical conditions [69]. Currently, research in this area is concentrated on two primary sensing modalities: radiographic chest imaging and digital pathological WSIs.

In the domain of chest radiography, the abundance of paired image-report datasets [70] enables VLMs to cultivate a generalized semantic understanding. By learning synchronized representations, these models can extrapolate knowledge from common ailments (e.g., generic pneumonia) to diagnose novel categories (e.g., COVID-19) [71,72]. To refine this zero-shot performance and mitigate critical misclassifications, several advanced training paradigms have been introduced. For example, self-supervised frameworks utilizing momentum distillation [43] and cross-attention modules for prompt alignment [44] have proven effective in standardizing diverse diagnostic expressions. Furthermore, when navigating multi-label clinical scenarios, the construction of similarity matrices through feature disentanglement can significantly boost recognition accuracy [45]. Other innovative strategies include multi-level contrastive pre-training to align key visual-textual features [73] and the decomposition of expert knowledge into multi-dimensional visual descriptors—such as texture and morphology—to facilitate precise cross-modal matching [74].

Conversely, zero-shot pathology diagnosis via WSIs [75] faces a profound “resolution mismatch”. While standard VLMs are typically pre-trained on low-resolution natural imagery, digital pathology sensors produce gigapixel-scale outputs (often exceeding 100,000 pixels). In real clinical practice, processing such ultra-high-resolution WSIs with standard transformer-based VLMs incurs prohibitive computational complexity. Because the memory footprint of self-attention mechanisms scales quadratically with sequence length, directly inputting WSI tokens frequently exceeds the VRAM limits of standard hospital-grade GPUs and results in unacceptable inference latency. To bridge this gap, architectural adaptations such as multi-instance learning have been deployed to reconstruct VLM frameworks for super-resolution analysis [76]. Additionally, the integration of residual feature connections and advanced token-merging optimization strategies within CLIP-based architectures allows for the efficient blending of pre-trained knowledge with newly acquired pathological features [77]. This not only optimizes diagnostic precision across numerous organ and tumor types but also drastically reduces the required fine-tuning duration. However, critically evaluating the negative results of these open-vocabulary diagnostic models reveals severe vulnerabilities, particularly concerning rare diseases. Because medical pre-training datasets are inherently imbalanced, generalized VLMs and specific models like BiomedCLIP often over-rely on statistical “shortcuts” learned from common pathologies [78]. Consequently, when prompted to identify underrepresented conditions—such as rare pediatric anomalies (e.g., esophageal strictures) or rare genetic tumors—these models frequently generate high rates of false positives [79]. This phenomenon, known as multi-modal hallucination, occurs because the models prioritize generating fluent, clinically plausible narratives over adhering strictly to ambiguous visual evidence [80]. Addressing these false positives and ensuring safe zero-shot inference for rare diseases remains a major bottleneck for the clinical deployment of VLMs.

To provide a highly intuitive and systematic cross-comparison of the aforementioned methodologies, Table 3 summarizes the quantitative performance of representative medical VLMs on benchmark diagnostic datasets across fine-tuning, few-shot, and zero-shot settings. This comparative analysis highlights the relative strengths of current architectures while also exposing certain evaluation limitations, such as the lack of rigorous baseline comparisons against peer pre-trained models.

### 4.2. VLMs for Medical Image Segmentation

The core applications of VLMs in medical image segmentation encompass visual-textual feature fusion, semi-supervised segmentation, zero-shot segmentation, and the transfer of 2D pre-trained models to 3D segmentation tasks.

#### 4.2.1. Fusion of Textual Prompts and Visual Sensor Features

In the realm of medical image segmentation, VLMs are predominantly deployed to fully integrate linguistic instructions with visual data. Here, text prompts serve as weak supervisory signals to systematically enhance the interpretation of complex outputs from medical imaging sensors [94]. This paradigm empowers clinicians to interact with diagnostic systems via natural language; for instance, a user query such as “localize small nodules within the left lung cavity” can automatically trigger targeted detection and segmentation. Furthermore, during report generation, the incorporation of textual prompts streamlines the extraction of critical diagnostic markers, significantly standardizing the final clinical narrative [31].

Current methodologies driving this cross-modal fusion generally follow two distinct trajectories: lightweight attention-based integration and large-scale pre-trained prompt adaptation [95].

Lightweight attention-based integration avoids the computational overhead of exhaustive fine-tuning on massive pre-trained networks. Instead, the primary research focus shifts toward the architectural design of prompt construction, optimal injection nodes, and customized attention mechanisms designed to precisely map textual targets to specific spatial regions captured by the sensors. Attention mechanisms have become indispensable for establishing robust language-to-visual correlations. For instance, bridging textual semantics with multi-level image features via cross-attention has proven highly effective in segmenting small-scale regions [23]. Beyond feature-level mapping, text-guided positional attention modules have been utilized to accurately align language with the spatial relationships of pixels, thereby refining localization accuracy [15]. In this context, integrating multi-scale contextual attention networks [96] can significantly reinforce spatial feature alignment for weakly supervised, prompt-driven segmentation. Moreover, leveraging lightweight architectures equipped with multi-attention mechanisms—similar to advanced YOLO variants [97]—offers a highly valuable reference for achieving efficient, real-time VLM-driven object detection and segmentation in resource-constrained clinical settings. Furthermore, attention mechanisms can be integrated with external domain expertise; as demonstrated by Zhang et al. [98], incorporating pathological knowledge into text-prompted weak supervision significantly enhances the reliability of generated pseudo-labels. Consequently, in complex or ambiguous clinical scenarios (such as defining infection boundaries or fuzzy pathological tissues), these textual cues act as vital supplementary signals to compensate for inherent visual ambiguities within the raw sensor data. The efficacy of these attention-based mechanisms is intricately tied to where the prompts are injected within the neural architecture. Since different network layers capture varying levels of feature abstraction—ranging from low-level sensor artifacts in shallow layers to high-level semantics in deep layers—researchers have strategically varied the point of integration. Depending on the target task, prompts have been successfully injected directly into the decoder [23], seamlessly positioned between the encoder and decoder [99], or embedded precisely at the network’s bottleneck layer [15].

Large-scale pre-trained prompt adaptation, conversely, leverages the immense prior knowledge of generalized VLMs to automatically synthesize diverse and complex prompt structures. To maximize the semantic depth of these prompts, multi-prompt fusion mechanisms (utilizing visual-text gated networks) [100] and bi-level graph matching strategies capable of capturing both word- and sentence-level semantics [34] have been engineered. Simultaneously, enhancing the modal adaptability between the prompts and the medical visual features remains a critical pursuit. To achieve this, conditional contrastive learning paradigms have been deployed to tighten cross-modal alignment [101]. For parameter efficiency, lightweight adaptation modules like VLSM-Adapter [102] have been introduced, enabling fine-tuning exclusively on specific adapters without perturbing the frozen pre-trained weights. Furthermore, explicitly embedding anatomical priors into textual prompts has proven to be a highly effective strategy for boosting segmentation precision across multi-class tumor architectures [103].

#### 4.2.2. Zero-Shot Paradigms for Unsupervised Medical Segmentation

Compared with medical image classification, segmentation annotation relies heavily on medical expert resources, and annotations for numerous anatomical structures remain scarce [104]. Zero-shot segmentation based on VLMs eliminates the need for additional training on novel categories, enabling direct application to the segmentation of unseen images and targets [105]. This technology is applicable to the image segmentation of rare lesions or emerging diseases, such as the segmentation of lesion ranges in rare bone tumors and the extraction of boundaries in infection areas of new infectious diseases. In emergency scenarios, it can quickly segment key regions such as hemorrhages and edema, providing timely support for the formulation of emergency treatment plans [106].

Dense pixel-level annotation of medical structures is a notoriously resource-intensive task, often constrained by the limited availability of radiological expertise. VLMs offer a compelling alternative through zero-shot segmentation, which enables the partitioning of visual signals from various sensors into semantic regions without task-specific training. Based on the scope of their generalization, current methodologies can be categorized into universal multi-modality frameworks and task-oriented specialized paradigms.

1. Universal Multi-Modality Frameworks: These models aim to achieve broad-spectrum segmentation across diverse imaging modalities (e.g., CT, MRI, and X-ray) and heterogeneous anatomical targets. The primary challenge lies in establishing a highly versatile cross-modal feature space. For instance, the integration of language-driven parameter generators allows models to capture complex semantic relationships between organs and tumors, facilitating the seamless inclusion of novel categories [107]. To enhance the precision of ROI (Region of Interest) localization within these universal systems, hybrid architectures like FluoroSAM [108] have been developed, combining the semantic strengths of CLIP with the high-fidelity segmentation capabilities of Segment Anything Model (SAM). Furthermore, the use of massive synthetic datasets (e.g., covering over 1.6 million radiographic sensor outputs) has proven effective in scaling the model’s ability to recognize hundreds of anatomical and non-anatomical targets [109].

2. Task-Oriented Specialized Paradigms: In contrast, these approaches focus on optimizing performance for specific clinical objectives by leveraging large-scale pre-trained priors. High-precision alignment is often achieved through sophisticated prompt engineering and feature decoupling. Specifically, frameworks such as Zept [110] utilize query decoupling to distinguish between static anatomical backgrounds and dynamic tumor regions, complemented by self-prompting mechanisms for adaptive feature refinement. For pathological analysis, where spatial complexity is significant, fusing linguistic cues with spatial information enables the flexible segmentation of intricate structures like renal tubules or cell nuclei [111]. Other strategies involve using SAM as a high-performance decoder, where target-aware heatmaps (generated via CAM-based methods) are utilized as visual prompts to guide the zero-shot segmentation process [112]. Despite these promising paradigms, zero-shot segmentation is not without failures in clinical practice. For instance, when applied directly to complex medical images without task-specific fine-tuning, generalist foundation models like SAM frequently struggle with low-contrast anatomical boundaries—such as distinguishing fuzzy ground-glass opacities in CT scans or obscure tumor margins in ultrasound. Furthermore, they often fail to delineate complex tubular topologies like retinal blood vessels [113]. In these negative scenarios, the severe domain shift between natural and medical images typically leads to severe over-segmentation or complete misses of target structures, highlighting that purely prompt-driven zero-shot methods still require substantial domain-specific adaptation to avoid clinical errors.

#### 4.2.3. Semi-Supervised Learning in Medical Image Segmentation

Semi-supervised learning plays a pivotal role in reducing the reliance of medical image segmentation tasks on large-scale labeled data. For instance, in tasks such as renal tumor segmentation [114], semi-supervised learning enables model training by combining a small volume of labeled data with a large amount of unlabeled data, thereby reducing the cost of data annotation.

Additionally, this approach is well-suited for multi-center research scenarios, where unlabeled data from different institutions can be integrated to enhance the generalization ability of the trained models [115]. To alleviate the reliance on extensive annotated datasets, VLMs have been increasingly integrated into semi-supervised medical image segmentation frameworks [116,117,118]. The fundamental paradigm relies on a dual-pronged strategy to maximize the utility of both limited labeled samples and abundant unlabeled data. Specifically, linguistic cues are actively exploited to guide and refine the generation of high-quality pseudo-labels for unannotated images. Concurrently, for the available labeled data, the supervised training phase is significantly fortified by aligning image-text contextual features and enhancing cross-modal interactions.

Several methodological advancements have been proposed to execute these semi-supervised strategies. For instance, the reliability of semi-supervised learning can be substantially improved by deeply fusing textual and visual information during the pseudo-labeling process [119]. To preserve fine-grained local details within this process, hybrid architectures such as LViT [120] integrate Convolutional Neural Networks with Transformers. By employing pixel-level attention and specialized language-vision loss functions, such models enable the direct text-driven optimization of unlabeled medical images. Furthermore, to mitigate the computational burden of model adaptation across diverse modalities, in-context learning paradigms have been introduced. Approaches like SegICL [121] leverage minimal image-mask pairs alongside text prompts to capture generalized contextual knowledge, thereby achieving versatile modal adaptation without the necessity of extensive retraining.

#### 4.2.4. 3D Medical Image Segmentation

Volumetric datasets acquired through sophisticated sensing modalities, such as high-resolution CT and MRI, are indispensable for understanding complex anatomical topographies. By providing depth-aware spatial context, 3D sensor outputs facilitate the capture of anatomical continuity and local geometric consistency [122]—elements that are often lost in planar projections. Within this framework, 3D imaging represents a continuous volumetric sampling paradigm [123], where the physical configuration of the sensor array and the slice intervals dictate the ultimate fidelity of the acquired clinical data.

Despite these diagnostic advantages, the processing of 3D volumes is hindered by the sheer density of voxels, which imposes a heavy computational burden on neural architectures. When deploying 3D-native VLMs in real clinical practice, the computational complexity scales cubically with spatial resolution. This results in massive GPU memory consumption and prolonged inference times, making real-time bedside analysis highly challenging. Consequently, models are often forced to rely on aggressive down-sampling or patch-based sliding window techniques, which inevitably risk losing vital global anatomical context. A systemic gap currently exists between foundational VLMs—which are predominantly optimized for 2D slices—and the volumetric domain, often resulting in significant performance degradation due to “cross-dimensional domain shift” during direct fine-tuning. To reconcile these discrepancies and enhance the 3D comprehension of VLMs, contemporary research focuses on two synergistic avenues: engineering robust transfer learning mechanisms to adapt 2D-pre-trained features to volumetric spaces, and aggregating large-scale, 3D-native medical datasets to foster native volumetric representation learning.

To overcome these cross-dimensional hurdles, architectural innovations such as Med3DInsight [46] have been developed to harmonize 3D volumetric embeddings with 2D multi-modal LLMs. By introducing slice-aware transformer mechanisms, this framework ensures a cohesive alignment between planar textual representations and the depth-dependent features of 3D sensor outputs. Beyond spatial alignment, the transition to high-resolution 3D analysis requires capturing intricate local details that standard 2D models might overlook. This complex adaptation can be facilitated by embedding clinical domain knowledge and employing specialized pre-training objectives—such as text-driven contrastive learning—to meticulously refine the volumetric signal representations [47]. Furthermore, to circumvent the critical scarcity of paired 3D image-text data, recent frameworks have leveraged LLMs to automatically synthesize clinically relevant textual descriptions directly from raw 3D inputs. These synthetically generated narratives subsequently serve as supervisory signals to guide the learning trajectory of 3D visual features [48].

Alongside architectural refinements, expanding the scale of 3D-native corpora is essential for enhancing model generalization. A significant contribution in this area is the construction of a comprehensive whole-body 3D medical dataset [124], which organizes over 22,000 volumetric images within a hierarchical anatomical knowledge tree. By integrating thousands of standardized clinical terms with large-scale 3D sensing data, this foundation-level training enables VLMs to achieve diagnostic performance that rivals or exceeds that of specialized, task-specific models.

### 4.3. Generation of Medical Image Reports

The transformation of raw signals acquired from imaging sensors into human-readable reports [125] was among the earliest implementations of multi-modal AI. Together with medical VQA, these systems facilitate the conversion of digitized visual data into finalized clinical narratives. This not only ensures the consistency of medical records but also substantially reduces the manual reporting workload faced by healthcare professionals in data-intensive environments [126].

#### 4.3.1. Integrating Contextual and Global Semantics

The synthesis of medical reports from high-resolution visual data is a highly complex sequence-generation task. Since clinical narratives are inherently lengthy and must often align with a patient’s historical records, merely detecting localized abnormalities is insufficient. To generate coherent and clinically accurate reports, models must maintain a delicate balance between global interpretation and contextual awareness. Global features are essential for providing the network with a macroscopic understanding of the entire anatomical landscape. Concurrently, contextual semantics prevent the isolated interpretation of specific lesions by actively grounding local features within their surrounding tissues and mapping them to appropriate diagnostic terminologies.

To effectively construct these intricate intra-image and textual relationships, several advanced topological frameworks have been proposed. One primary strategy involves memory-augmented and knowledge-driven architectures. For example, deep contextual correlations among generated words can be captured via attention-enhanced relational memory networks (e.g., AERMNet [25]), which utilize two-layer LSTMs to guarantee narrative coherence. Similarly, expert domain knowledge can be explicitly injected into the feature space; transformer-based frameworks [127] have successfully fused visual grid representations with graph convolutions and auxiliary language modules to comprehensively enrich contextual awareness.

Another effective methodology focuses on selective attention and feature recalibration to align textual and visual streams. By applying semantic attention during the visual encoding phase, models can differentially weight critical anatomical regions, which is subsequently complemented by nearest-neighbor search strategies in the decoder to retrieve relevant contextual text [128]. Furthermore, dynamic visual recalibration modules [129] have been introduced to explicitly extract salient local features, ensuring that the contextual alignment between the identified lesions and the generated long-text descriptions remains highly precise and logically consistent.

#### 4.3.2. Fine-Grained Cross-Modal Mapping

Translating complex visual signals acquired from medical sensors into highly accurate professional reports relies on robust cross-modal correlations. To precisely capture subtle localized abnormalities and map them to specialized clinical terminologies, vision-language models must move beyond macro-level fusion and achieve fine-grained alignment. Current paradigms addressing this intricate requirement can be broadly classified into three fundamental strategies: localized semantic matching, hierarchical multi-scale alignment, and anatomy-driven grounding.

Localized semantic matching focuses on establishing a strict one-to-one mapping between specific visual patches and exact textual phrases. Since standard cosine similarity metrics are often inadequate for capturing nuanced local interactions, researchers have introduced intermediate modalities to bridge the gap. For instance, phrasebooks (PhraseAug [130]) and adaptive patch extraction modules enabling bidirectional cyclic generation [131] have been successfully utilized. Furthermore, precise cross-modal entity mapping can be achieved through supervised fine-tuning driven by image-text matching scores [21], or by integrating radiological knowledge graphs with multi-head attention mechanisms (e.g., FgKF [132]).

Rather than relying on a single level of feature extraction, hierarchical multi-scale alignment progressively bridges the modalities from global semantics down to detailed word-level interactions. This stratification is typically modeled along linguistic constructs (e.g., report-to-sentence-to-word) or organized by specific disease categories. Under this paradigm, sentence topics have been deployed as intermediate supervisory signals for contrastive learning [133]. Similarly, mechanisms guided by disease labels [134,135] and dynamic fusion architectures like AGFNet [136] have been proposed to adaptively balance global contextual features and local details based on whether the analyzed sample exhibits abnormalities.

Recognizing that diagnostic images represent actual physical structures captured by medical sensors, anatomy-driven grounding explicitly embeds spatial and physiological priors into the matching process. This approach effectively resolves the common limitation of generic models that overlook clinical topological specificity. To emulate the natural diagnostic workflow of radiologists, models can explicitly isolate anatomical regions of interest via object detection frameworks (e.g., RGRG [137]) or utilize specific organ segmentation masks for feature association [138]. Other prominent strategies involve integrating instance-level expert knowledge [139] or employing weakly supervised activation maps (such as Grad-CAM) to precisely pinpoint the origin of the described pathologies [140]. Additionally, as clinical applications scale globally, ensuring the accurate alignment of multilingual clinical narratives becomes imperative. Recent parameter-efficient fine-tuning frameworks, which unify cross-lingual multimodal event extraction [141], offer an efficient adaptation strategy to address this gap in multilingual medical report generation using VLMs.

### 4.4. Construction of Medical Multi-Task General Foundation Models

Medical multi-task general foundation models leverage the general feature construction capability of VLMs to adapt to downstream tasks. These models enable one-stop comprehensive medical image analysis, capable of simultaneously completing multiple tasks such as image classification, segmentation, lesion detection, and report generation [142].

The research sub-fields of medical multi-task general large models mainly include the following five aspects.

#### 4.4.1. Contrastive Learning

Image-text contrastive learning (ITC) jointly optimizes visual and textual encoders to align paired medical images and clinical texts in a shared embedding space, thus bridging the cross-modal semantic gap [29]. However, pure instance-level (global) contrastive alignment may miss the small yet critical lesion cues in medical images and the fine-grained descriptions in reports [49]. As a result, recent studies emphasize local and multi-granular alignment, enforcing matching constraints within each image-report pair at the level of regions, words, and sentences.

Representative approaches include GLoRIA [50], which adds a local contrastive objective to align word embeddings with attention-weighted image-region features and provides attention-based interpretability; LRCLR [143] further selects disease-related regions via self-attention and contextualizes them with a cross-modal transformer before computing local contrastive losses. Since word-level alignment can be context-insufficient and prone to mismatches [20], Liao et al. [144] improve region-sentence association by maximizing local mutual information. LOVT [145] combines instance-level ITC with region–sentence local contrast, allowing multiple positives per region and encouraging consistency across neighboring regions, which benefits locality-sensitive tasks such as segmentation and detection. BioViL-T [146] guides visual attention with textual semantics for better interpretability. MGCA [83] introduces prototype clustering and extends alignment from instance-level to token-level and disease-level to enhance fine-grained discrimination, while IMITATE [20] performs hierarchical vision–language alignment by matching multi-level chest X-ray features to descriptive and impression sections of structured reports. Other methods (e.g., MedCLIP [82], Liu et al. [87]) adopt semantic-aware sample selection mechanisms to address the misclassifications of semantically similar pairs.

#### 4.4.2. Masked Predictive Learning

It randomly masks a portion of image/text tokens and trains the model to reconstruct the missing content, enabling robust cross-modal representation learning in a self-supervised manner. In medical VLMs, common forms include Masked Image Modeling (MIM), Masked Language Modeling (MLM), and multi-modal masked modeling, where masked local regions are recovered or predicted with textual cues to strengthen fine-grained structural modeling and local-global alignment [51]. Beyond that, multi-modal masked autoencoding frameworks such as M3AE [147] unify image-text learning under reconstruction objectives, improving multi-task transfer and robustness to missing/noisy modalities; MCG-Net [19] further introduces chief complaint-guided multi-modal masked content modeling for chest image classification tasks.

#### 4.4.3. Matching Prediction

Matching prediction (MP) formulates image-text matching as a classification problem, with the most common instantiation being Image-Text Matching (ITM). ITM predicts whether an input image-text pair is matched (positive) or mismatched (negative): if the image and text come from the same paired instance, the model outputs a high matching probability; otherwise, a low probability, typically optimized with binary cross-entropy [148]. For medical VLMs, MP/ITM is often combined with ITC and masked prediction objectives to build hybrid training signals that jointly encourage discriminative alignment and fine-grained semantic modeling [91,149]. However, hybrid objectives also introduce additional optimization complexity and potential inter-task gradient interference, making training efficiency under performance constraints an open challenge.

Beyond standard ITM, several studies incorporate knowledge-enhanced classification supervision during pre-training by leveraging auxiliary dataset labels or generating scalable “entity/disease presence” labels via external labelers and medical knowledge bases. For example, KAD [91] samples positive/negative entities from UMLS [150] to train a knowledge encoder, and augments chest X-ray–report alignment with entity presence prediction over frequent corpus entities. MedKLIP [85] extracts medical triplets from clinical reports and encodes them with domain knowledge, jointly optimizing contrastive and classification losses by treating triplet values as labels. UniBrain [151] extends such knowledge-enhanced classification objectives to MRI-based image–text representation learning, using a Transformer decoder that queries aligned MRI features with disease descriptions to predict disease presence, thereby learning more diagnosis-oriented cross-modal representations.

#### 4.4.4. Hybrid-Objective Pre-Training

Although contrastive learning, masked prediction, and matching prediction have shown strong performance in medical vision-language pre-training, each objective has its own limitations. Contrastive and matching-based methods may suffer from missed findings, while masking-based modeling often lacks strong zero-shot capability. To exploit complementary strengths, many studies adopt hybrid-objective pre-training, where the overall loss is typically formulated as a weighted sum of task-specific losses. However, multi-objective training increases optimization complexity and may induce inter-task interference, making training efficiency under performance constraints an open challenge.

1. Masked prediction + contrastive learning: The two objectives are naturally complementary: contrastive learning enhances discriminative alignment via explicit positive/negative separation, whereas masking promotes fine-grained representation learning through reconstruction. PMC-CLIP [81] augments global contrastive alignment with visually prompted MLM to strengthen cross-modal interaction. Zhang et al. [152] integrate alignment into a joint image–text reconstruction framework with a memory-enhanced fusion module. Silva et al. [153] extend MLM with multi-modal contrastive learning using a RealFormer-based encoder [154]. PRIOR [155] combines global/local alignment with cross-modal conditional reconstruction and introduces prototype vectors for sentence features to reduce reliance on syntactic details. In addition, some works initialize encoders with unimodal MLM or unimodal contrastive learning (alignment across augmentations within the same modality), e.g., Liu et al. [88] and BioViL [92], before applying cross-modal contrastive losses.

A key challenge is that masking may remove critical local cues, making masked image–text alignment difficult [156], and applying contrastive learning directly to masked inputs can degrade due to misalignment [157]. Many approaches therefore optimize masking and contrastive objectives in different stages, or introduce mitigation strategies such as dynamically reweighting contrastive losses based on masked patch locations (e.g., MACO [157]), or reconstructing masked visual features before contrastive alignment [158]. Nevertheless, the improvements are often limited, calling for stronger joint optimization mechanisms.

2. Masked prediction + matching prediction: Contrastive learning is often performed with limited fusion and may not capture rich cross-modal interactions, whereas matching prediction is applied to fused multi-modal embeddings and better cross-modal interactions. UWOX [159] enables unified pre-training over paired and unpaired data with multi-scale MIM and MP losses. MedViLL [148] pretrains with MLM and ITM and proposes a bidirectional autoregressive self-attention masking mechanism. Zhang et al. [149] inject domain knowledge via UMLS and introduce knowledge-guided similarity alignment, knowledge-aware fusion, and knowledge-aware ITM and MIM objectives.

3. Masked prediction + contrastive learning + matching prediction: Some studies combine all three to jointly support alignment, interaction modeling, and fine-grained semantics. PTUnifier [160] uses prompts to handle paired/unpaired inputs and jointly optimizes MLM, ITM, and ITC. Li et al. [161] employ MIM, MLM, ITC, and ITM on medical image-caption data and achieve strong results on medical VQA. MUMC [162] further incorporates unimodal and multi-modal contrastive losses alongside MLM/ITM, using a MoCo-style momentum encoder for unimodal contrast and random patch masking as augmentation.

#### 4.4.5. Other Variants

Beyond hybrid objectives, other targets such as generation, clustering, distillation, and regularization have also been explored. Seibold et al. [163] propose a distillation-based contrastive loss with both global and local contrast; the local loss is adapted from MIL-NCE [164] under the assumption that clinically relevant information lies in a subset of report sentences. REFERS [84] optimizes report generation conditioned on image embeddings, while Tier [165] regularizes sparse token–region correspondence. For medical VQA, Zhan et al. [166] propose UnICLAM, achieving deeper alignment and ex-ante interpretability via progressive soft parameter sharing and adversarial masking augmentation.

## 5. Existing Challenges and Future Directions

Despite the remarkable progress of VLMs in medical imaging, the transition from experimental prototypes to clinical deployment faces substantial hurdles. Based on recent advancements, we summarize the core challenges and potential technical solutions into three major directions.

### 5.1. From “Static Alignment” to Interactive Clinical Diagnostic Agents

**Challenge:** Existing methods often struggle with fine-grained semantic alignment, where subtle pathological details are missed due to global feature matching. Static alignment fails to capture the dynamic, logical flow of real clinical diagnosis (“Symptom → Evidence → Diagnosis”).

**Future Direction:** Integrating LLMs to build interactive clinical diagnostic agents.

- Deep Visual-Linguistic Alignment via Instruction Tuning: Evolving models from simple classifiers into agents capable of complex VQA through multi-modal instruction tuning.
- Chain-of-Thought Reasoning: Explicitly modeling the clinical reasoning process to emulate a doctor’s logic.
- Human-in-the-Loop Interaction: Transforming AI from a “black box” predictor into a responsive assistant via point-and-click or interactive textual corrections.

### 5.2. From “Correlation” to Trustworthy and Causal Medical AI

**Challenge:** Medical VLMs face issues regarding fairness, bias, and lack of interpretability. Reliance on statistical correlations leads to “shortcut learning” and demographic biases, making it dangerous to rely on AI for high-stakes clinical decisions.

**Future Direction:** Establishing trustworthy AI via causal inference and uncertainty quantification.

- Causal Representation Learning: Integrating structural causal models to distinguish genuine pathological features from confounding background noise via counterfactual reasoning.
- Uncertainty Quantification: Integrating Bayesian deep learning or evidence theory to output uncertainty estimates alongside diagnoses.
- Prototype-based Interpretability: Enhancing interpretability by retrieving “Case Prototypes” from historical cases to provide evidence-based, clinically verifiable decision support.

### 5.3. From “Image-Text Alignment” to Multi-Source Sensor-Driven Intelligence

**Challenge:** Current medical VLMs are primarily optimized for high-resolution, static imaging modalities (e.g., CT, MRI), often treating the data as isolated snapshots. However, they frequently overlook the rich temporal and physiological information captured by diverse medical sensors, such as real-time ECG streams, PPG signals, and wearable motion detectors. This creates a “sensing silo,” where the fragmented data from heterogeneous sensors cannot be effectively synchronized with visual findings and clinical narratives. Consequently, existing models struggle to provide a longitudinal and holistic assessment of patient health, particularly in scenarios requiring continuous monitoring.

**Future Direction:** Developing unified frameworks that harmonize multi-source sensor streams with vision-language representations for continuous clinical monitoring and decision support.

- Temporal Signal-to-Semantic Mapping: Engineering universal encoders and specialized tokenizers capable of mapping raw 1D physiological sensor signals into discrete semantic tokens. This allows Transformer-based VLMs to “read” heart rate variability or neural oscillations alongside medical images within a unified embedding space.
- Dynamic Multi-Sensor Synchronicity: Implementing dynamic alignment mechanisms to fuse high-frequency sensor data with low-frequency imaging snapshots. By capturing the temporal correlations between acute physiological fluctuations and structural pathological evolutions, models can achieve a more comprehensive understanding of disease progression. To computationally support such dynamic optimization, integrating advanced geometric optimization algorithms—such as pairwise Frank–Wolfe methods [167]—can significantly enhance the efficiency of real-time multimodal alignment under geometric constraints, thereby providing crucial algorithmic support for the dynamic optimization of sensor-driven intelligence.
- Intelligent Edge Sensing Platforms: Transitioning VLMs toward lightweight implementations for internet of medical things devices. This enables real-time diagnostic reasoning directly on wearable sensors, facilitating immediate clinical alerts while preserving data privacy.

## 6. Conclusions

VLMs have undeniably established a new consensus within the field of medical image analysis, with their application trajectory rapidly evolving from basic cross-modal feature fusion to the widespread deployment of multi-task general foundation models. However, beyond merely summarizing this established technological progression, this review distinguishes itself through two original, framework-level contributions that aim to reshape how VLMs are conceptualized and deployed in clinical settings.

First, rather than treating VLMs conventionally as generic multi-modal feature extractors—a common perspective in existing literature—this study uniquely distills their underlying mechanisms into seven core operational principles. By systematically mapping these distinct principles (e.g., fine-grained interpretability, prompt-driven weak supervision, and zero-shot reasoning) directly to specific clinical downstream tasks, a structured taxonomy is provided to demystify VLM operations for clinical practitioners.

Second, this review transcends the traditional “image-text alignment” paradigm by explicitly incorporating the physical constraints of medical hardware. By analyzing the specific data characteristics of distinct sensor modalities (e.g., MRI RF coils, CT detectors), a novel, forward-looking framework is formulated: the transition towards “multi-source sensor-driven intelligence”. By identifying critical bottlenecks such as data scarcity, static reasoning, and correlational biases, this study maps out strategic future directions aimed at promoting the clinical translation of VLM-based technologies—evolving them from static diagnostic tools into trustworthy, dynamic, and sensor-aware clinical diagnostic agents.

## Figures and Tables

**Table 1:** Research method and literature selection criteria.

Items	Description
Search date	Updated continuously up to the revision phase (May 2026)
Sources	Web of Science, PubMed, IEEE Xplore, Scopus, arXiv, and Google Scholar
Specific Queries	Title/Abstract combined Boolean logic: ("Vision-Language Model" OR "VLM" OR "Vision-Language Foundation Model") AND ("Medical Image Analysis" OR "Clinical Imaging" OR "Medical Diagnosis" OR "Radiology") AND ("Multi-Modal" OR "Zero-Shot" OR "Few-Shot" OR "Weakly-Supervised")
Filters	Publication Type: Peer-reviewed Journal Articles, Conference Proceedings, and Preprints. Language: English only.
Period covered	2020–2026 (including Early Access publications and Preprints)
Inclusion criteria	1. Peer-reviewed articles and high-impact Preprints published in English; 2. Studies presenting novel VLM architectures or applications; 3. Empirical validation on medical imaging datasets; 4. Focus on multi-modal (vision-language) approaches.
Exclusion criteria	1. Non-English publications; 2. Purely uni-modal approaches (vision-only or language-only); 3. Studies lacking empirical validation; 4. Conference abstracts without full-text availability.
Selection process	Selection jointly performed by all authors following PRISMA flow.

**Table 2:** Applications and characteristics of VLM in medical imaging.

Principle	Definition	Key Technologies	Representative Methods	Highlights
1. Multi-Modal Fusion	Integrating visual and textual features from medical images and clinical narratives to enhance diagnostic accuracy.	Cross-modal attention; Feature alignment; Multi-scale fusion.	ViLa-MIL [33]; Berthop [22]; Bi-VLGM [34]	Comprehensive information integration.
2. Fine-Grained Interpretability	Providing transparent decision-making through visual explanations and concept bottleneck models.	Grad-CAM; Concept bottleneck layers; Knowledge graphs.	RC-TPL [35]; Concept filtering [36]; Nodule-CLIP [37]	Enhanced clinical trust.
3. Weakly-Supervised Learning	Leveraging unlabeled data with text prompts as weak supervision signals.	Prompt learning; Contrastive training.	ViLaCo [38]; Contrastive cross-modal pre-training [39]; MedPrompt [40]	Reduced annotation burden.
4. Few-Shot Learning	Adapting to new tasks with minimal labeled examples.	Meta-learning; Prompt engineering.	FlexR [41]; Two-level prompt learning [42]; MedPrompt [40]	Fast adaptation.
5. Zero-Shot Learning	Performing inference on unseen categories without task-specific training.	Contrastive learning; Semantic alignment.	Medical X-VL [43]; Carzero [44]; Feature disentanglement [45]	No training required for new categories.
6. Domain Transfer	Adapting models from 2D to 3D domains and across imaging modalities.	Transfer learning; 2D-3D feature alignment.	Med3DInsight [46]; T3D [47]; GTGM [48]	Leverages large 2D pre-trained models.
7. Foundation Models	Large-scale pre-trained models for multiple medical tasks.	Contrastive learning; Masked predictive learning; Matching prediction.	ConVIRT [49]; GLoRIA [50]; MRM [51]; MCG-Net [19]	Strong generalization.

**Table 3:** Comparison of representative medical VLMs on diagnostic tasks. The best result is bolded.

Setting	Method	Year	Dataset	Performance (%)	Remarks
Fine-tuning	PMC-CLIP [81]	2023	MedMNIST	**AUC = 99.02** (Pneumonia) **AUC = 94.56** (Breast) **AUC = 93.41** (Derma)	Limited baselines; lacks direct comparison with peer VLM pre-training models.
	GLoRIA [50]	2021	ChestX-ray14	AUC = 83.8	-
	ConVIRT [49]	2022	ChestX-ray14	AUC = 84.2	-
	MedCLIP [82]	2022	ChestX-ray14	AUC = 83.8	-
	MGCA [83]	2022	ChestX-ray14	AUC = 84.4	-
	REFERS [84]	2022	ChestX-ray14	AUC = 83.7	-
	MedKLIP [85]	2023	ChestX-ray14	AUC = 84.5	-
	M-FLAG [86]	2023	ChestX-ray14	AUC = 84.0	-
	MRM [51]	2023	ChestX-ray14	AUC = 85.3	-
	SAT [87]	2023	ChestX-ray14	AUC = 83.7	-
	MAVL [74]	2024	ChestX-ray14	AUC = 84.7	-
	MCG-Net [19]	2025	ChestX-ray14	**AUC = 86.4**	-
	Med-MLLM [88]	2023	COVIDx-CXR-2	**AUC = 98.4**	-
Few-shot	TIMNet [39]	2021	Mendeley-V2	**ACC ≈ 87.0** (1-shot)	Limited baselines; lacks direct comparison with peer VLMs.
	FlexR [41]	2024	Localized pathology	**AUC = 77.0** (5-shot)	Limited baselines; lacks direct comparison with peer VLMs.
	ViLa-MIL [33]	2024	TCGA-Lung	**AUC = 74.7** (16-shot)	Primarily compared with MIL/self-supervised methods; lacks VLM baseline comparisons.
	PoLe [68]	2024	Pneumonia	**ACC ≈ 74.0** (16-shot)	Evaluated solely against the baseline CLIP model.
Zero-shot	GLoRIA [50]	2021	CheXpert	AUC = 75.0	-
	MedCLIP [82]	2022	CheXpert	AUC = 74.4	-
	CheXzero [89]	2022	CheXpert	AUC = 88.9	-
	BiomedCLIP [90]	2023	CheXpert	AUC = 67.7	-
	MedKLIP [85]	2023	CheXpert	AUC = 87.9	-
	KAD [91]	2023	CheXpert	AUC = 90.5	-
	BioViL [92]	2023	CheXpert	AUC = 78.9	-
	CARZero [44]	2024	CheXpert	**AUC = 92.3**	-
	PLIP [93]	2023	DigestPath	**F1 = 0.832**	Evaluated primarily against the baseline CLIP model.

AUC: Area Under the Receiver Operating Characteristic Curve; ACC: Accuracy; F1: F1-score.

## Data Availability

The data presented in this study are available upon request.

## Abbreviations

The following abbreviations are used in this manuscript:

VLM	Vision-Language Model
AI	Artificial Intelligence
LLM	Large Language Model
VQA	Visual Question Answering
WSI	Whole Slide Image
ITC	Image-text contrastive learning
MIM	Masked Image Modeling
MLM	Masked Language Modeling
MP	Matching Prediction
ITM	Image-Text Matching
